# Induction of Tumor Necrosis Factor and Interleukin-6 mRNA in Human Cytotrophoblast Cells Exposed to Lipopolysaccharide

**DOI:** 10.1155/S1064744994000311

**Published:** 1994

**Authors:** Bernard Gonik, Jacob Rachmilewitz, Avraham Hochberg, Ran Goshen, Nathan de-Groot

**Affiliations:** 1 Department of Obstetrics, Gynecology, and Reproductive Sciences University of Texas Medical School Houston TX USA; 2 the Department of Biological Chemistry the Institute for Life Sciences at the Hebrew University Jerusalem Israel; 3 Department of Obstetrics and Gynecology Wayne State University School of Medicine Grace Hospital, 6071 W. Outer Drive Detroit MI 48235 USA

## Abstract

*Objective:* The cytokines tumor necrosis factor (TNF) and interleukin-6 (IL-6) have previously been identified in placental tissue and are known to be mediators of infection-associated induction of the host immune system. This study was undertaken to better characterize the in vitro regulation of these cytokines in cytotrophoblast cells when challenged with the bacterial product lipopolysaccharide (LPS).

*Methods:* Term placentas were freshly collected, digested with trypsin/DNase, and subjected to Percoll gradient centrifugation to isolate cytotrophoblasts. Either immediately or after overnight incubation, LPS (1 μg/ml) or media alone was added to the cell cultures for 0, 1, 2, 4, 8, 24, 48, and 72 h. Total cellular RNA was isolated by the guanidinium thiocyanate/cesium chloride methodology. RNA samples were run on 1% agarose-formaldehyde gels and subsequently transferred to nylon filters. Blots were hybridized with the appropriate P32-radiolabeled probe.

*Results:* In non-LPS-treated cells, minimal amounts of TNF mRNA could be detected at zero time, or throughout the incubation periods. Conversely, LPS exposure resulted in detectable signal starting at 1 h and peaking at 2 h after the addition of LPS. Overnight incubation gave stronger TNF signals in the LPS stimulated cells, although the kinetics of this response remained similar to zero time exposure. IL-6 was likewise minimally expressed at zero time, although non-stimulated cell cultures demonstrated progressive increases in mRNA expression which was maximal at 16 h after plating. LPS further augmented the transcription of IL-6 mRNA, with peak signals seen at 4 h after LPS stimulation. Again, overnight incubation of the cytotrophoblasts increased baseline and LPS-induced IL-6 mRNA responses. Long-term constant exposure of cells to LPS did not demonstrate any evidence of prolonged signaling. LPS did not alter mRNA expression of the placental gene H19 or the oncogene FOS.

*Conclusions:* These data demonstrate the induction of TNF and IL-6 mRNA in cytotrophoblast cells with LPS. These transcriptional events are kinetically distinct and short term in nature. Overnight incubation accentuates the TNF and IL-6 mRNA signal and allows for an augmented response to LPS.


Infectious Diseases in Obstetrics and Gynecology 2:3–9 (1994)
© 1994 Wiley-Liss, Inc.

### Induction of Tumor Necrosis Factor and Interleukin-6 mRNA in Human Cytotrophoblast Cells Exposed to Lipopolysaccharide

**Bernard Gonik, Jacob Rachmilewitz, Avraham Hochberg, Ran Goshen, and Nathan de-Groot**

*Department of Obstetrics, Gynecology, and Reproductive Sciences, University of Texas Medical School, Houston, TX (B.G.); and the Department of Biological Chemistry, the Institute for Life Sciences at the Hebrew University, Jerusalem, Israel (J.R., A.H., R.G., N.d.-G.)*

### Abstract

*Objective:* The cytokines tumor necrosis factor (TNF) and interleukin-6 (IL-6) have previously been identified in placental tissue and are known to be mediators of infection-associated induction of the host immune system. This study was undertaken to better characterize the in vitro regulation of these cytokines in cytotrophoblast cells when challenged with the bacterial product lipopolysaccharide (LPS).

*Methods:* Term placentas were freshly collected, digested with trypsin/DNase, and subjected to Percoll gradient centrifugation to isolate cytotrophoblasts. Either immediately or after overnight incubation, LPS (1 μg/ml) or media alone was added to the cell cultures for 0, 1, 2, 4, 8, 24, 48, and 72 h. Total cellular RNA was isolated by the guanidinium thiocyanate/cesium chloride methodology. RNA samples were run on 1% agarose-formaldehyde gels and subsequently transferred to nylon filters. Blots were hybridized with the appropriate P32-radiolabeled probe.

*Results:* In non-LPS-treated cells, minimal amounts of TNF mRNA could be detected at zero time, or throughout the incubation periods. Conversely, LPS exposure resulted in detectable signal starting at 1 h and peaking at 2 h after the addition of LPS. Overnight incubation gave stronger TNF signals in the LPS-stimulated cells, although the kinetics of this response remained similar to zero time exposure. IL-6 was likewise minimally expressed at zero time, although non-stimulated cell cultures demonstrated progressive increases in mRNA expression which was maximal at 16 h after plating. LPS further augmented the transcription of IL-6 mRNA, with peak signals seen at 4 h after LPS stimulation. Again, overnight incubation of the cytotrophoblasts increased baseline and LPS-induced IL-6 mRNA responses. Long-term constant exposure of cells to LPS did not demonstrate any evidence of prolonged signaling. LPS did not alter mRNA expression of the placental gene H19 or the oncogene FOS.

*Conclusions:* These data demonstrate the induction of TNF and IL-6 mRNA in cytotrophoblast cells with LPS. These transcriptional events are kinetically distinct and short term in nature. Overnight incubation accentuates the TNF and IL-6 mRNA signal and allows for an augmented response to LPS. © 1994 Wiley-Liss, Inc.

Key words

Pregnancy, placenta, immunology, infection

It is well recognized that the placenta is a rich source for a variety of cytokines. Tumor necrosis factor (TNF) and interleukin-6 (IL-6) have been previously identified within placental tissues, specifically associated with cytotrophoblasts, and are known mediators of infection-associated induction of the immune system.[1–5] Since the placenta functions as an immunologic as well as mechanical barrier to vertically transmitted infection, these cytokines may play an important role in mediating this immune response.[6] However, data are currently lacking which adequately define the in vitro cy-

**Address correspondence/reprint requests to Dr. Bernard Gonik, Department of Obstetrics and Gynecology, Wayne State University, School of Medicine, Grace Hospital, 6071 W. Outer Drive, Detroit, MI 48235.**
**Presented at the Society of Perinatal Obstetricians, Las Vegas, NV, 1994.**

Received March 11, 1994

Accepted May 10, 1994

Basic Science Article totrophoblast response to exposure to various infectious agents or their products.

This report represents data which better characterize the in vitro transcriptional regulation of TNF and IL-6 in cytotrophoblast cells challenged with the bacterial product lipopolysaccharide (LPS). Herein we define both the short- and long-term kinetics of this response. In addition, since short-term in vitro culturing of cytotrophoblasts is known to alter cellular differentiation and therefore transcriptional events,[7] we examined the influence of time after culturing as it relates to LPS induction of both TNF and IL-6 transcription. Lastly, to see if LPS caused a general or specific induction of cytotrophoblast mRNA, we examined the effect of LPS on the non-cytokine placental H19 gene and the oncogene FOS.

### SUBJECTS AND METHODS

Cytotrophoblasts were purified from freshly acquired, term placentas from uncomplicated pregnancies, according to the method of Gileadi and associates.[8] Briefly, after the maternal decidual surface was sharply dissected away, approximately 200 g of minced coteledonary tissue, scraped free of blood vessels and connective tissue, was digested for 30 min at 37°C in modified Hank's balanced salt solution (HBSS) containing 0.1% trypsin (Sigma, St. Louis, MO) and 0.66 mg/g DNase (Sigma). Subsequently, cytotrophoblasts were obtained by collecting the 35–55% fractions of a Percoll gradient centrifugation. After being counted (Coulter Electronic, Harpenden, Hertfordshire, UK), cells were plated in sterile flasks containing Dulbecco's modified Eagle's medium (DMEM) and F12 modified media (Gibco, Paisley, UK) with 10% fetal calf serum (FCS) and antibiotics. The *Staphylococcus albus* phagocytosis assay was utilized in separate experiments to demonstrate that less than 1% of the cultured cells were of the monocyte/macrophage lineage (data not shown).

Culture flasks containing between 20 and $40 \times 10^6$ cytotrophoblasts were exposed to 1 μg/ml of LPS or media alone, either immediately after plating or after an overnight preincubation period. In some experiments, short-term TNF and IL-6 kinetics (after LPS exposure for 0, 1, 2, 4, 8, and 24 h) were compared for the above 2 preincubation periods. Long-term TNF and IL-6 kinetic studies, using freshly incubated cells exposed to either LPS or media alone, were carried out at 0, 24, 48, and 72 h. For the experiments involving the H19 and FOS probes, we studied only freshly collected cells, which were incubated with or without LPS for up to 24 h.

Total cellular RNA was isolated from cells by the guanidinium thiocyanate/cesium chloride methodology.[9] Lithium chloride and ethanol precipitations were used to purify the samples. RNA was quantitatively assessed by ultraviolet spectrophotometry. A comparison of the optical density (OD) 260 and 280 values was used to assess RNA quality, along with mini-gel preparations.

Northern blot analyses were carried out by first loading each well of a 1% agarose-formaldehyde gel with 8 μg of RNA sample. After electrophoresis, samples were transferred to Hybond nylon filters (Amersham Corporation, Amersham, UK). Blots were stained with methylene blue to confirm equal amounts of RNA in each lane. Hybridization was carried out with specific P32-labeled oligonucleotide probes (Oncogene Science, Uniondale, NY) or from random prime-labeled probes prepared from appropriate clones. Signal was detected by autoradiography using AGFA Curix film at −80°C. At least 3 separate experiments were carried out for each comparison.

### RESULTS

In Figure 1 is shown the short-time curve for the mRNA expression of TNF with (+) and without (−) LPS exposure. As can be seen, incubation with LPS leads to TNF mRNA expression, beginning at 1 h and peaking at approximately 2 h. This transcriptional response to LPS is relatively short-lived, with most of the expression gone by 8 h.

The long-time curve for TNF expression (Fig. 2) demonstrates, after prolonged autoradiography, low-grade expression of the TNF mRNA at 24, 48, and 72 h. However, this expression is no different for the cytotrophoblasts incubated with LPS or with media alone.

Figure 3 examines TNF mRNA expression, with and without LPS exposure, for 2 different preincubation periods. In the top portion of this figure are data for cells freshly isolated and immediately tested for TNF response to LPS. On the

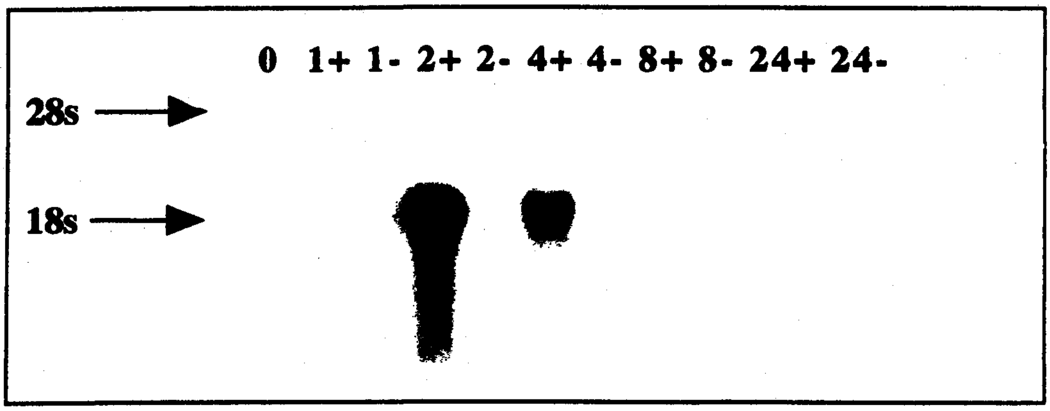

Fig. 1. Short-time curve for LPS induction of TNF mRNA in human cytotrophoblast cells.

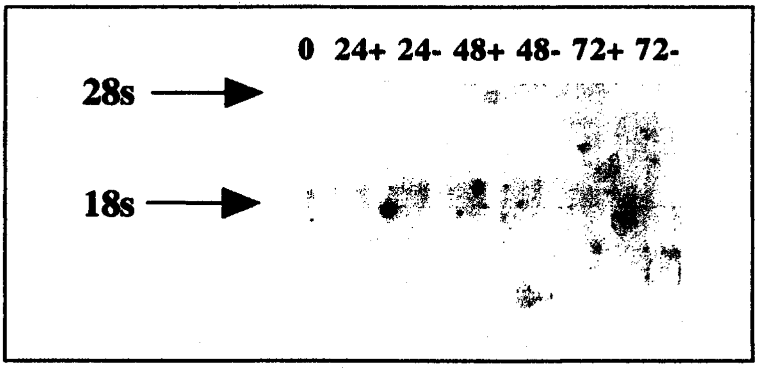

Fig. 2. Long-time curve for LPS induction of TNF mRNA in human cytotrophoblast cells.

bottom is an autoradiograph of TNF mRNA expression for cells preincubated overnight, prior to LPS exposure. These data are from a single filter, loaded with equal amounts of sample RNA from the individual experiments. As can be seen, although the kinetics for mRNA TNF expression are the same, preincubation of the cytotrophoblasts overnight results in a more pronounced expression of the TNF mRNA for each comparable time point.

In Figures 4–6 are shown representative data for the effect of LPS on IL-6 transcription. Again, LPS exposure results in a clearly defined transcriptional response for IL-6. Peak expression occurs at approximately 4 h and, unlike TNF, continues to be strongly expressed through the 24-h time period (Fig. 4). Note that IL-6 expression can be seen from cultured cytotrophoblasts even without LPS exposure. LPS, however, accentuates this mRNA response.

As with TNF, long-term continuous in vitro LPS stimulation of cytotrophoblasts has minimal effects on IL-6 mRNA expression (Fig. 5). Although mRNA expression of IL-6 continues through the 72-h time point, there is very little difference between those cells incubated with LPS-spiked media and those incubated in media without LPS.

In Figure 6 is shown the effect of overnight preincubation of cytotrophoblasts, prior to LPS exposure, on IL-6 expression. As with TNF, overnight preincubation results in a more pronounced mRNA expression of IL-6 for each comparable time point.

Figures 7 and 8 are representative autoradiographs examining the effect of LPS on the non-cytokine genes H19 and FOS. Time points for these experiments were chosen based on previously published kinetic studies,[7] using optimal expression periods. For H19 (Fig. 7), LPS has no demonstrable effect on gene expression up to 24 h.

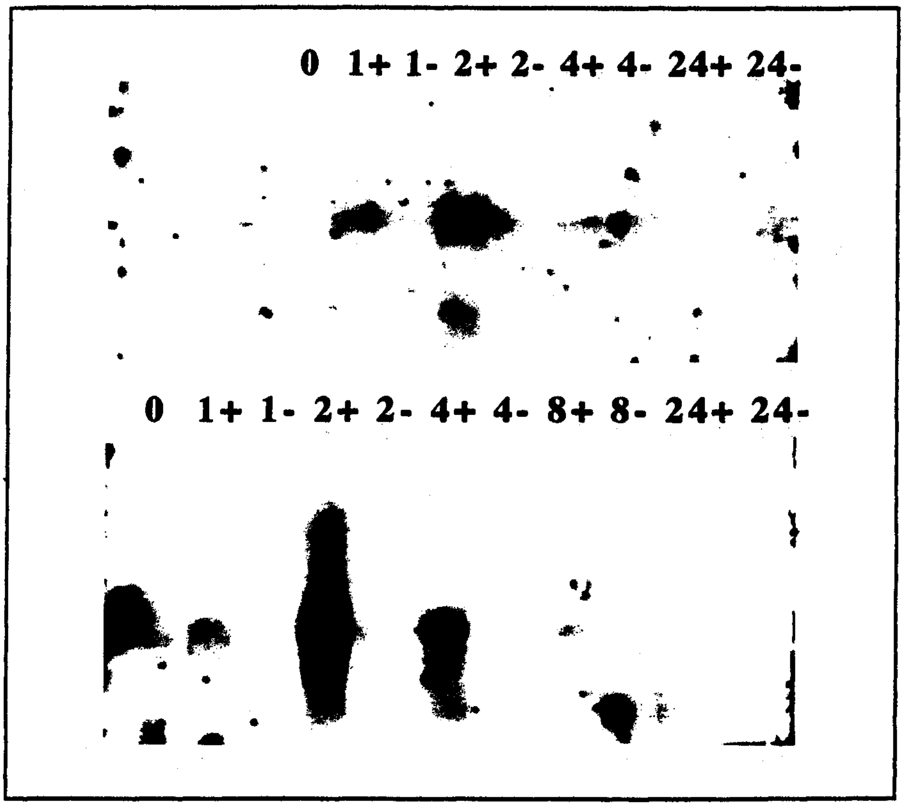

Fig. 3. Effect of varying the preincubation period [immediate (**above**); overnight (**below**)] prior to LPS induction of TNF mRNA in human cytotrophoblast cells. Note that the 8-h time point is missing in the top experiment.

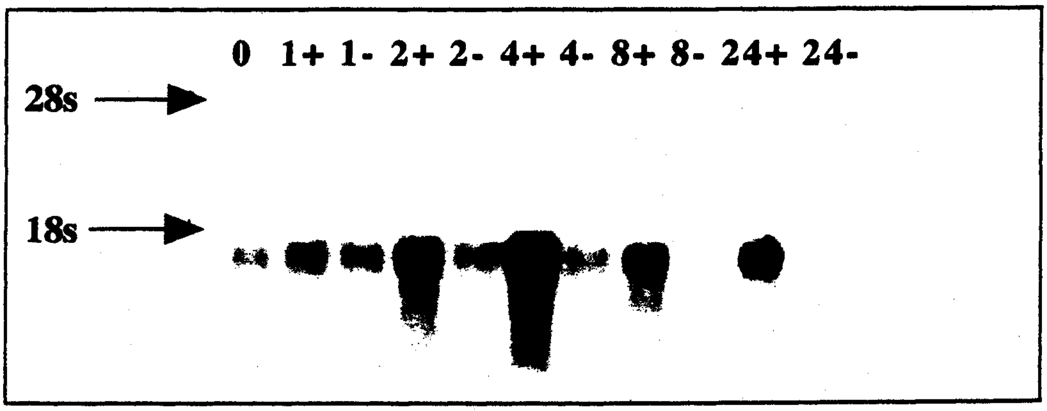

Fig. 4. Short-time curve for LPS induction of IL-6 mRNA in human cytotrophoblast cells.

This is also true for the oncogene FOS (Fig. 8), which has a much shorter peak transcriptional period than H19, but is similarly unaffected by LPS.

### DISCUSSION

The placenta performs a variety of critical functions during pregnancy including regulation of nutrient transport, hormonal secretion, and immunoregulation to prevent fetal rejection.[10] Additionally, recent data have suggested that the placenta may also be involved in the protection of the fetus against a variety of infectious agents by both mechanical and functional mechanisms.[4,6,11–15] Specifically, local immune activation of the placenta by invading

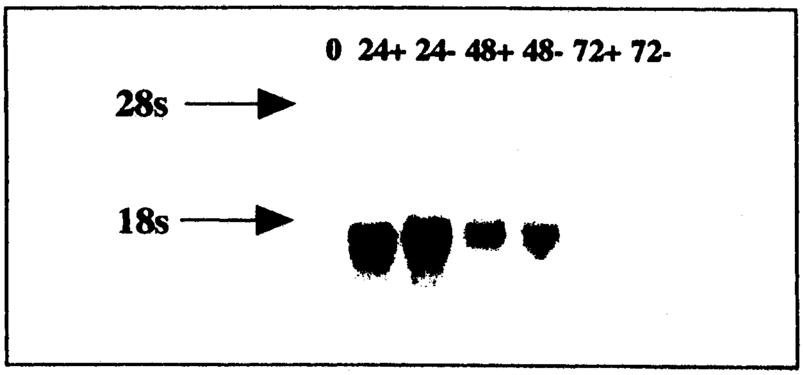

Fig. 5. Long-time curve for LPS induction of IL-6 mRNA in human cytotrophoblast cells.

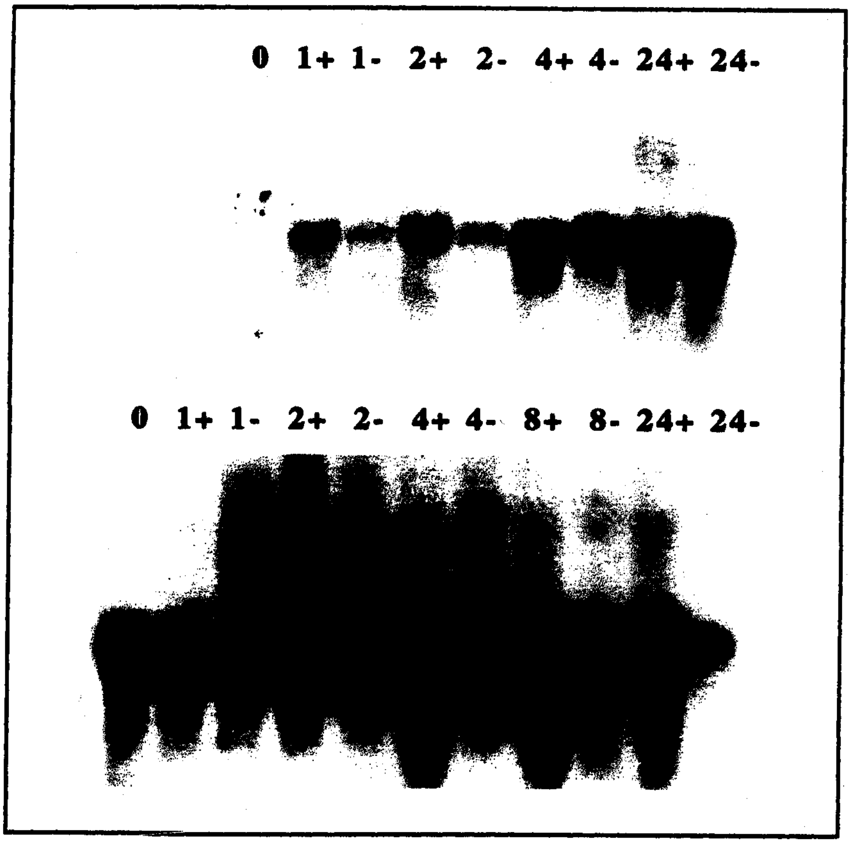

Fig. 6. Effect of varying the preincubation period [immediate (above); overnight (below)] prior to LPS induction of IL-6 mRNA in human cytotrophoblast cells. Note that the 8-h time point is missing in the top experiment.

pathogens (or their byproducts) is a likely means by which these tissues respond to this challenge in an attempt to protect the fetus.

Previous investigators[1–5,16] have demonstrated the production of various cytokines and their receptors by placental tissues, including the syncytio- and cytotrophoblasts, and resident Hofbauer cells. Cytokines are known mediators of the inflammatory response to infection, but details of their regulation in this setting have yet to be thoroughly defined. In this study, we examined the kinetics of TNF and IL-6 transcription using a short-term in vitro cell culture model of human cytotrophoblasts. Our data demonstrate a selective induction of TNF

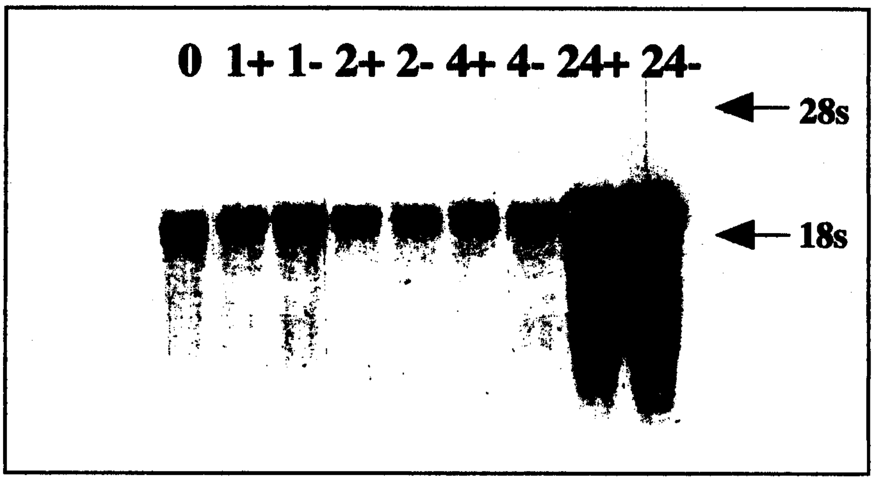

Fig. 7. Lack of effect of LPS exposure on HI9 mRNA expression in human cytotrophoblast cells.

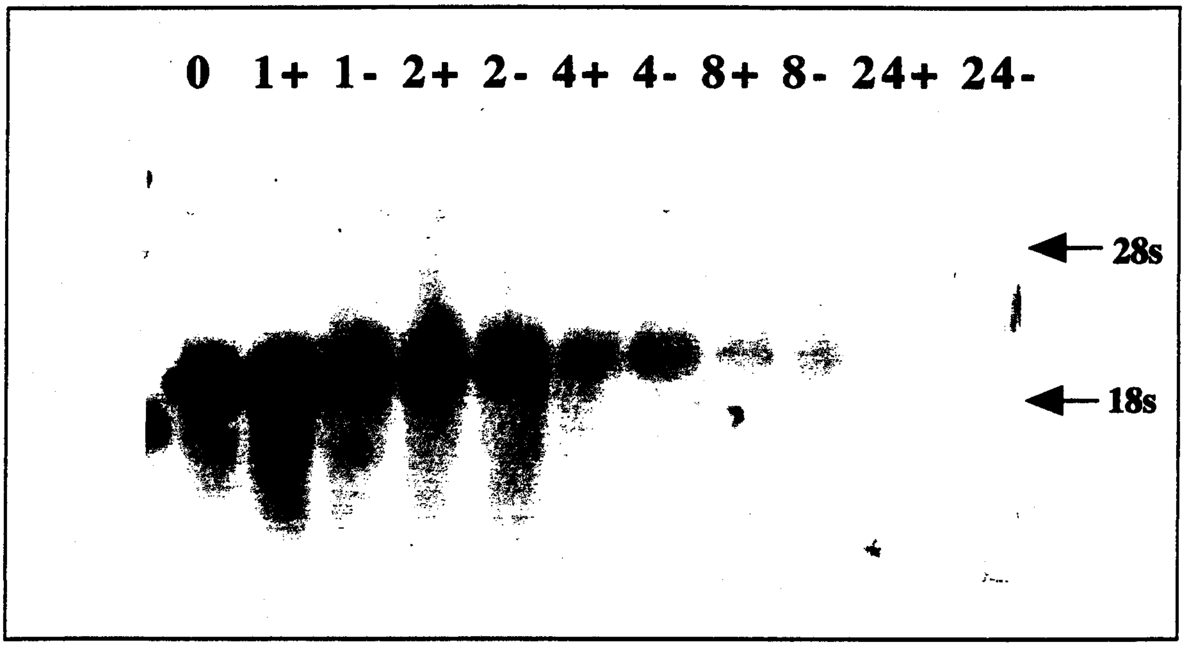

Fig. 8. Lack of effect of LPS exposure on FOS mRNA expression in human cytotrophoblast cells.

and IL-6 mRNA with exposure of cytotrophoblasts to the bacterial product LPS. These transcriptional events are kinetically distinct and short term in nature. The exact role of these cytokines still requires clarification, since, in addition to regulation of the inflammatory response, TNF and IL-6 are also involved in modulating cell growth and differentiation.[17,18] In this regard, TNF alpha has been recently shown to induce human chorionic gonadotropin in human trophoblast cells via an IL-6-dependent mechanism.[2] Likewise, immune signaling at the maternal-fetal interface influences trophoblastic cell differentiation.[19] Once this role has been better defined, future studies can be directed at immunoregulation of these cellular components in hopes of attenuating infectious morbidity.[20]

Of interest are our findings that in vitro culturing of term cytotrophoblasts leads to an altered response to LPS induction of TNF and IL-6. Overnight incubation resulted in an accentuation of the TNF and IL-6 mRNA signals, suggesting an augmented transcriptional response to LPS. These results are in keeping with other investigations[7,21,22] which have shown that cytotrophoblasts undergo rapid differentiation in culture, with substantial variations in mRNA expression, protein synthesis, and cell morphology. The dynamics of this in vitro system must therefore be kept in mind when interpreting and comparing these and other data.

### ACKNOWLEDGMENTS

This work was supported by the Berlex Foundation International Research Fellowship Award.

### REFERENCES

1. Chen HL, Yang Y, Hu XL, Yelavarthi KK, Fishback JL, Hunt JS: Tumor necrosis factor alpha RNA and protein are present in human placental and uterine cells at early and late stages of gestation. Am J Pathol 139:327–335, 1991.
2. Li Y, Matsuzaki N, Masuhiro K, Kameda T, Taniguchi T, Saji F, Yone K, Tanizawa O: Trophoblast-derived tumor necrosis factor-α induces release of human chorionic gonadotropin using interleukin-6 (IL-6) and IL-6-receptor-dependent system in the normal human trophoblasts. J Clin Endocrinol Metab 74:184–191, 1992.
3. Kameda T, Matsuzaki N, Sawai K, Okada T, Saji F, Matsuda T, Hirano T, Kishimoto T, Tanizawa O: Production of interleukin-6 by normal human trophoblast. Placenta 11:205–213, 1990.
4. Casey ML, Cox SM, Beutler B, Milewich L, MacDonald PC: Cachectin/tumor necrosis factor-α formation in human decidua, potential role of cytokines in infection-induced preterm labor. J Clin Invest 83:430–436, 1989.
5. Yang Y, Yelavarthi KK, Chen HL, Pace JL, Terranova PF, Hunt JS: Molecular, biochemical, and functional characteristics of tumor necrosis factor-alpha produced by human placental cytotrophoblastic cells. J Immunol 150: 5614–5624, 1993.
6. Benirschke K, Kaufmann P: Infectious diseases. In: Pathology of the Human Placenta. New York: Springer-Verlag, pp 542–635, 1990.
7. Rachmilewitz J, Gonik B, Goshen R, Ariel J, Schneider T, de-Groot N, Hochberg A: Intermediate cells during cytotrophoblast differentiation in vitro. Cell Growth Differ 4:395–402, 1993.
8. Gileadi O, Schneider T, Chebath J, de-Groot N, Hochberg AA: The expression of proto-oncogenes and 2′,5′ oligoadenylate synthetase in differentiating human trophoblastic cells in vitro. In Mochizuki M, Hussa R (eds): Placental Protein Hormones. New York: Elsevier, pp 251–260, 1988.
9. McDonald RJ, Calvin HS, Przyblgla AE, Chirgwin JM: Isolation of RNA using guanidinium salts. In Berger SL, Kimmel AR (eds): Methods in Enzymology. Academic Press, New York: pp 224–226, 1987.
10. Ringler GE, Strauss JF III: In vitro systems for the study of human placental endocrine function. Endocr Rev 11: 105–123, 1990.
11. Jauniaux E, Nessman C, Inbert C: Morphological aspects of the placenta in HIV pregnancies. Placenta 9:633–642, 1988.
12. Chandwani S, Greco MA, Mittal K: Pathology and human immunodeficiency virus expression in placentas of seropositive women. Infect Dis 163:1134–1138, 1991.
13. Romero R, Manogue KR, Mitchell MD, Wu YK, Oyarzun E, Hobbins JC, Cerami A: Infection and labor. IV. Cachectin-tumor necrosis factor in the amniotic fluid of women with intraamniotic infection and preterm labor. Am J Obstet Gynecol 161:336–341, 1989.
14. Oliveira LHS, Fonseca MEF, DeBonis M: Placental phagocytic cells infected with herpes simplex type 2 and echovirus type 19: Virological and ultrastructural aspects. Placenta 13:405–416, 1992.
15. Taniguchi T, Matsuzaki N, Kameda T, Shimoya K, Jo T, Saji F, Tanizawa O: The enhanced production of placental interleukin-1 during labor and intrauterine infection. Am J Obstet Gynecol 165:131–137, 1991.
16. Yelavarthi KK, Hunt JS: Analysis of p60 and p80 tumor necrosis factor-α receptor messenger RNA and protein in human placentas. Am J Pathol 143:1131–1141, 1993.
17. Fiers W: Tumor necrosis factor, characterization at the molecular, cellular and in vivo level. Fed Eur Biochem Soc 285:199–212, 1991.
18. Van Snick J: Interleukin-6: An overview. Annu Rev Immunol 8:253–278, 1990.
19. Wegmann TG, Guilbert LJ: Immune signaling at the maternal-fetal interface and trophoblast differentiation. Dev Comp Immunol 16:425–430, 1992.
20. Gonik B, Loo L, Reuben J, Cowles T, Helfgott A, Harris A, Doyle M: Placenta natural killer cell cytotoxicity in human immunodeficiency virus infected parturients (abstract). Am J Obstet Gynecol 166:290, 1992.
21. Gileadi O, Schneider T, de-Groot N, Chebath J, Revel M, Hochberg AA: Interferon beta-2 in differentiating human cytotrophoblast. Trophoblast Res 5:281–295, 1991.
22. Kliman HJ, Feinman MA, Strauss JF III: Differentiation of human cytotrophoblast into syncytiotrophoblast in culture. Trophoblast Res 2:407–421, 1987.
